# Middle East Respiratory Syndrome Coronavirus during Pregnancy, Abu Dhabi, United Arab Emirates, 2013

**DOI:** 10.3201/eid2203.151049

**Published:** 2016-03

**Authors:** Asim Malik, Karim Medhat El Masry, Mini Ravi, Falak Sayed

**Affiliations:** Mafraq Hospital, Abu Dhabi, United Arab Emirates

**Keywords:** MERS-CoV, Middle East respiratory syndrome coronavirus, pregnancy, WHO, World Health Organization, UAE, United Arab Emirates, Abu Dhabi, viruses, fatal outcome, influenza-like illness, surveillance, treatment

## Abstract

As of June 19, 2015, the World Health Organization had received 1,338 notifications of laboratory-confirmed infection with Middle East respiratory syndrome coronavirus (MERS-CoV). Little is known about the course of or treatment for MERS-CoV in pregnant women. We report a fatal case of MERS-CoV in a pregnant woman administered combination ribavirin–peginterferon-α therapy.

As of June 19, 2015, a total of 1,338 laboratory-confirmed cases of Middle East respiratory syndrome coronavirus (MERS-CoV) and 475 associated deaths had been reported to the World Health Organization (http://www.who.int/csr/disease/coronavirus_infections/risk-assessment-19june2015/en/). Payne et al. (*1*) reported stillbirth in a pregnant patient for whom MERS-CoV serologic testing was retrospectively positive, suggesting that surveillance for and treatment of MERS-CoV in pregnant women may differ from that for nonpregnant persons. We report a fatal case of MERS-CoV in a pregnant woman administered ribavirin–peginterferon-α therapy.

## Case Report

On November 19, 2013, a 32-year-old woman residing in Abu-Dhabi, United Arab Emirates, sought medical care for fever and back pain of 4 days’ duration. The woman, a school teacher from Jordan, was 32 weeks pregnant; she reported 3 earlier pregnancies (2 live births) and no concurrent conditions. Emergency department (ED) and obstetric physicians suspected urinary tract infection. The patient declined admission but returned to the ED on November 22 with worsening fever, cough, and shortness of breath. She denied recent travel, sick contacts, or animal exposure within the previous 2 weeks. Lung examination results were within normal limits; the patient had no signs of active labor or fetal distress. Chest computed tomography scan revealed bilateral consolidation; pulmonary embolus was not seen.

The patient was admitted to the medical unit with suspected community-acquired pneumonia. Ceftriaxone and azithromycin were initiated. Her condition deteriorated, and on November 23, she was transferred to the intensive care unit (ICU) because of respiratory failure and hypotension. On November 24, acute respiratory distress syndrome developed, requiring respiratory and hemodynamic support. Empiric oseltamivir and vancomycin were added to the treatment regimen. Later that day, the baby was delivered by caesarean section because the patient was persistently hypoxemic while on maximal ventilator support. Transient improvement in oxygenation was noted after the delivery. The newborn, who was noted to be healthy and had Apgar scores of 6 and 8 at 1 and 5 minutes, respectively, had no contact with the mother after birth.

Nasopharyngeal aspirate samples were tested for influenza A(H1N1)pdm09 virus and MERS-CoV by real-time reverse transcription PCR (*2*), and multiple other laboratory and culture tests were conducted (Table). Most yielded negative results, but on November 25, the regional laboratory reported the MERS-CoV real-time reverse transcription PCR results were positive. Laboratory testing was done by qualitative assay, using the 2012 novel human CoV (human coronavirus–Erasmus Medical Center). The assay, performed according to a previously described method (*3*), contains reagents and enzyme for specific amplification of the region upstream of the envelope gene in the CoV genome. 

**Table:** Laboratory and imaging investigation for a pregnant patient with MERS-CoV infection, Abu Dhabi, United Arab Emirates, 2013*

**Test**	**Finding**						
	22 Nov	24 Nov	25 Nov	26 Nov	27 Nov	28 Nov	29 Nov
**Urine culture**						Neg	Neg
**Blood culture**	Neg	Neg				Neg	Neg
**High vaginal swab sample**	Neg						
**Sputum culture**		Neg					
**Tracheal aspirate culture**							Neg
**MDR screen**		Neg					
**MRSA screen**		Neg					
**Legionella**		Neg					
**Influenza A**		Neg					
**Influenza B**		Neg					
**H1N1 PCR**		Neg					
**MERS-CoV rRT-PCR, specimen**		1 Neg, 1 Pos, C_t_ 34.10, naso aspirate	Pos, C_t_ 22.02, tracheal aspirate		Pos, tracheal aspirate		Neg, tracheal aspirate
**Leukocytes, × 10^9^ cells/L**	5.03	6.76	21.57	18.22	10.51	15.69	9.52
**Chest imaging, modality**	Bilateral consolidation, contrast CT		Bilateral consolidation, radiograph	Resolving consolidation and edema, radiograph		Worsening edema, radiograph	Improved edema and consolidation, radiograph

*Specimen processing was performed by using the EZ1 Advanced XL instrument (QIAGEN, Hilden, Germany) with the LightCycler (Roche Diagnostics, Indianapolis, IN, USA) or Rotor-Gene (QIAGEN) instrument for automated amplification and detection. The detection limit of the assay is 3.4 copies per reaction. Blank cells indicate no testing was done on that day. CT, computed tomography; C_t_, cycle threshold; H1N1, influenza A(H1N1)pdm09 virus; MDR, multidrug resistance; MERS-CoV, Middle East respiratory syndrome; MRSA, methicillin-resistant *Staphylococcus aureus*; naso aspirate, nasopharyngeal aspirate; Neg, negative; Pos, positive; rRT-PCR, real-time reverse transcription PCR.

On November 26, oral ribavirin (400 mg and 600 mg morning and evening, respectively) and subcutaneous peginterferon-α (180 µg 1×/wk) were initiated. On November 27, ribavirin was increased to 1,200 mg every 8 hours, and meropenem was begun. Septic shock developed in the patient, requiring maximal vasopressors and ventilator support. Despite intensive support, the patient’s condition continued to deteriorate; she died on December 2. Cultures of blood, tracheal aspirate, and urine obtained on the day of death showed no growth; a chest radiograph revealed improvement in pulmonary edema and consolidation.

On November 21, MERS-CoV pneumonia developed in the patient’s husband. He had no concurrent conditions and fully recovered after receiving an antimicrobial drug regimen similar to his wife’s at a different facility. The husband subsequently reported that he and his wife had visited a cattle farm (goats, sheep, and camels) 10 days before becoming sick (*4*) (Figure) but had not consumed camel meat or milk. In addition, a mild cough without fever or other symptoms developed in the patient’s 8-year-old son; MERS-CoV PCR testing of nasopharyngeal aspirate from the boy was positive. He recovered uneventfully without intervention. The younger sibling and newborn remained asymptomatic and tested negative for MERS-CoV.

**Figure:**
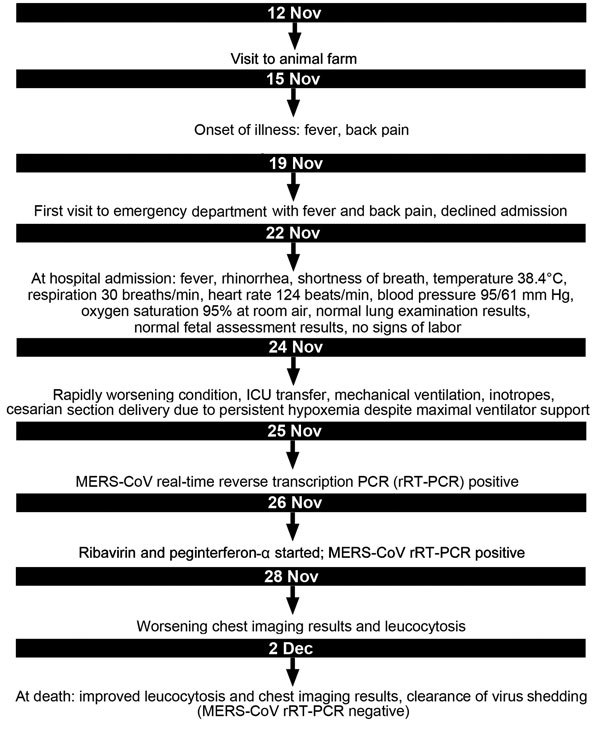
Timeline of clinical events in a pregnant patient with Middle East respiratory syndrome coronavirus (MERS-CoV) infection, Abu Dhabi, United Arab Emirates, 2013. ICU, intensive care unit.

Patients infected with MERS-CoV typically show signs of respiratory illness (*2*) and sometimes diarrhea. Complications include acute renal failure and acute respiratory distress syndrome with shock. Immunocompromised patients may have atypical signs and symptoms. Furthermore, several issues are relevant to MERS-CoV and other infectious diseases acquired during pregnancy: 1) pregnancy is associated with immunologic changes that may alter susceptibility to and severity of infectious diseases; 2) the effects of infection upon the fetus are not fully understood; and 3) prophylaxis and treatment appropriate for the general population might not be appropriate for pregnant women (*5*–*8*). When the pregnant patient in our study sought medical care, she had atypical symptoms (fever and back pain, followed later by cough and shortness of breath). It is unclear if the delay in initiating antimicrobial therapy may have contributed to the fatal outcome.

MERS-CoV infection developed in 2 of the patient’s 4 other family members, an outcome compatible with the description of other small clusters among household members and close contacts. The spectrum of illness and symptomatology among the affected family is also noteworthy: the young child was mildly sick, whereas the pregnant mother died. This discrepancy in disease severity correlates with findings from other reports (*9*,*10*).

No MERS-CoV transmission was documented between the patient and hospital staff; thus, the staff’s use of contact and droplet precautions, including airborne precautions while performing aerosol-generating procedures, seems to have been effective (*11*,*12*). Of note, these precautions were implemented after the patient was transferred to the ICU. Thirty-six hospital personnel were screened: 11 ED, 13 obstetrics, 1 operating room, and 11 ICU staff. No symptoms developed, and all staff tested negative for MERS-CoV infection. The lack of cross transmission in the exposed healthcare workers before implementation of protective measures supports the benefit of using standard precautions and the fact that transmission of MERS-CoV between humans remains limited (*13*).

Although, MERS-CoV infection and pregnancy were a fatal combination for the patient in our study, virus shedding ceased in the patient during therapy with ribavirin and peginterferon-α. Knowledge about the use of these drugs is limited; thus, further studies are needed to understand the possible safety, efficacy, and optimal dosage and duration of this regimen. Few data exist regarding the use of ribavirin in pregnant humans; however, the drug is generally contraindicated in pregnancy (*14*,*15*) because of evidence of teratogenic and embryocidal effects in animal studies. The potential role of cyclosporine, intravenous immune globulin, and high-frequency ventilation for treatment of MERS-CoV during pregnancy needs evaluation.

After the baby was delivered, the patient showed transient improvement, with improved oxygenation, followed by progressive worsening of clinical status and death. It is unclear whether there is a pattern of delayed release of chemokines and activation of inflammatory cascades leading to delayed worsening of the clinical condition. The patient was maintained on broad-spectrum antibacterial drugs with excellent pharmacokinetics. All cultures and screening for resistant pathogens remained negative, making it unlikely that the patient succumbed to a superimposed bacterial infection.

## Conclusions

Pregnant women who seek medical care for pneumonia, influenza-like illness, or sepsis on the Arabian Peninsula may benefit from screening for MERS-CoV to ensure early diagnosis and management of this sometimes fatal disease. The immunologic and chemokine response to the infection needs close examination to help define the potential therapeutic role of antiinflammatory agents in this disease.

MERS-CoV infection and pregnancy were a fatal combination in this case. Death occurred despite treatment with a combined ribavirin and interferon regimen and despite clearance of virus shedding and radiographic evidence of improvement at death. Thus, this regimen needs to be further studied in pregnant patients with MERS-CoV infection.
